# Seven Years of Vitrified Blastocyst Transfers: Comparison of 3 Preparation Protocols at a Single ART Center

**DOI:** 10.3389/fendo.2020.00346

**Published:** 2020-05-29

**Authors:** Paolo Emanuele Levi Setti, Federico Cirillo, Raffaella De Cesare, Emanuela Morenghi, Valentina Canevisio, Camilla Ronchetti, Annamaria Baggiani, Antonella Smeraldi, Elena Albani, Pasquale Patrizio

**Affiliations:** ^1^Division of Gynecology and Reproductive Medicine, Department of Gynecology, Fertility Center, Humanitas Clinical and Research Center (IRCCS), Milan, Italy; ^2^Biostatistics Unit, Humanitas Clinical and Research Center (IRCCS), Milan, Italy; ^3^Yale University Fertility Center, New Haven, CT, USA; ^4^Yale University Fertility Center, New Haven, CT, United States

**Keywords:** blastocyst warming, endometrial preparation, clinical pregnancy rate, live birth rate, endometrial receptivity

## Abstract

**Introduction:** Frozen–thawed embryo transfers (FET) have become a standard practice to increase cumulative pregnancy rates, however, the choice of the best preparation protocol remains a matter of debate.

**Design:** Retrospective analysis of clinical pregnancy (CPR) and live birth rate (LBR) of FET in natural cycles (NC-FET), modified natural cycles with hCG-triggered ovulation (mNC-FET), and hormonal artificial replacement (AR-FET).

**Materials and Methods:** For natural cycles, patients were monitored by ultrasound to evaluate the dominant follicle and by urinary LH kits (NC-FET). When the endometrial thickness reached at least 7 mm and the dominant follicle 16–20 mm, hCG was administered in absence of urinary LH surge (mNC-FET). Embryo thawing and transfer was planned 7 days after LH surge or hCG administration. For the AR-FET, oral estradiol valerate was administered from day 2 of menstrual cycle until endometrial thickness reached at least 7 mm and transfer was planned after 5 days of vaginal progesterone start. Only single vitrified blastocyst transfers were included.

**Results:** In total 2,895 transfers were performed of which 561 (19.4%) carried out with NC-FET, 1,749 (60.4%) with mNC-FET and 585 (20.2%) with AR-FET. CPRs were 32.62, 43.05, and 37.26%, respectively. LBR were 24.06, 33.56, and 25.81%, respectively. A statistically significant (*p* < 0.001) higher LBR for mNC-FET vs. NC-FET (OR 0.49–0.78) and AR-FET (OR 0.47–0.74) was observed. A higher ectopic pregnancy rate (*p* = 0.002) was observed in NC-FET (3.28%) than in AR-FET (1.83%) and mNC-FET (0.40%). A higher abortion rate (*p* = 0.031) in pregnancies <12 weeks was observed in AR-FET (27.52%) than in NC-FET (19.67%) and in mNC-FET (19.39%). At *Post hoc* analysis only female age (OR 0.91–0.95), antimullerian hormone (AMH) (OR 1.01–1.07) and mNC-FET (OR 1.39–1.98) were statically significant prognostic factors for LBRs.

**Conclusions:** These results demonstrate a superior CPR and LBR following FET in hCG-triggered ovulation cycles compared to NC and AR-FET, a higher ectopic pregnancy rate in NC-FET and a higher abortion rate in pregnancies <12 weeks in AR-FET. However, these data need to be confirmed in randomized and prospective studies before definitive conclusions can be drawn.

**Clinicaltrials.gov ID: NCT03581422**

## Introduction

Embryo cryopreservation is a common and indispensable tool used during *in vitro* Fertilization (IVF) treatments to cryopreserve excess embryos. This cost-effective and safe procedure prevents the risk of multiple pregnancies (1–3), ovarian hyper stimulation syndrome (4, 5) and reduce the need of repeating ovarian stimulation cycles if the fresh transfer is not successful. Furthermore, it allows to delay embryo transfer in cases in which endometrial preparation is not optimal (6, 7) or when there is a premature progesterone rise, while also warranting an increase in cumulative pregnancy rates per oocyte retrieval (8). In addition, this policy has now also been extended to cycles with pre-implantation genetic testing (9, 10).

Efficient cryobiology procedures and optimal embryo survival rates are essential for a successful frozen–thawed embryo transfer (FET) program (11, 12) as is an adequate synchronization between the endometrium and the embryonic developmental stages. Despite an increasing number of FET cycles, no consensus has been reached regarding the best replacement strategy for optimal endometrial preparation (13–17).

Three types of protocols are commonly used for endometrial synchronization during FET: natural cycles (NC-FET), modified natural cycles (mNC-FET) with human Chorionic Gonadotropin (hCG) triggering, and artificial replacement cycles (AR-FET) in which the endometrium is prepared using estrogen and progesterone. Each of these regimens can be modified by addition of progesterone for luteal support (in NC or mNC cycles) or by changing the dosage of medications used (in AR cycles) (18).

Another less commonly employed FET preparation strategy is with a mild exogenous ovarian stimulation either with gonadotropins or clomiphene citrate to increase serum estrogen and potentially enhance endometrial receptivity; however, recently this protocol lost favor due to its lack of effectiveness (19). In our center, normally ovulating patients or patients presenting a contraindication to estrogen therapies, FET is commonly performed in natural cycle regimens (20). The latter implies less medical intervention but requires the detection and documentation of ovulation by numerous monitoring visits even in women with regular menstrual cycles. Furthermore, the date of embryo transfer cannot be planned in advance, which may represent a problem for centers that do not operate 7 days a week (18). A limited number of retrospective studies (16, 21, 22) and randomized controlled trials (RCT) (23, 24) compared NC-FET and mNC-FET, but they either did not demonstrate significant differences in clinical outcomes or showed conflicting results.

The present retrospective study aimed at comparing the effectiveness of exclusive NC-FET vs. mNC-FET and AR-FET on Clinical Pregnancy Rates (CPR) and Live Birth Rates (LBR).

## Materials and Methods

### Ethical Aspects

All data were collected using an exclusive internal web-based database fully described elsewhere (25), with patients' data safeguarded by an advanced threat prevention, enterprise-class encryption, and any user needs periodical password renewal. Patients had consented in writing to the use of their anonymized medical records for research purposes. Since both conditions were met, this study had expedited review and approval. The study was registered in Clinicaltrial.com before full variables extraction and statistical analysis (ID: NCT03581422). The study was approved by the Independent Ethical Committee of the Humanitas Institutional Clinic (Milan, Italy). Consent was obtained from each patient after full explanation of the purpose and nature of all procedures used.

### Inclusion and Exclusion Criteria

This retrospective comparative analysis was carried out between 2011 and 2017 and included a total of 2,895 FET cycles. To limit potential confounders, only patients who underwent single blastocyst transfers with vitrified/rewarmed day 5 or day 6 blastocysts were included. Pre-implantation genetic test cycles were excluded (10).

### Intervention Description

The primary endpoint of the study was comparing clinical pregnancy rate (CPR) and live birth rate (LBR) of pure natural cycle frozen–thawed embryo transfer (NC-FET) vs. natural cycle frozen–thawed embryo transfer with hCG-triggered ovulation (mNC-FET) and hormonal artificial replacement (AR-FET). Secondary endpoint was pregnancy outcome.

The decision to assign patients to natural or modified natural cycle or an artificial replacement cycle was determined by the patient's ovulatory status, menstrual cycle regularity and presence of contraindications to estrogen supplementation such as, for example, previous intolerance. Natural cycle was considered as first choice, due to good patient's compliance and because it does not require any or few additional medications. Freeze all cycles and polycystic ovary patients were also included.

In natural cycles patients had serial transvaginal ultrasound monitoring (TU) starting between cycle day 8–12 to detect the dominant follicle and assessing endometrial development (26). Patients were instructed to start monitoring for urinary Luteinizing Hormone (LH) testing when a follicle with a mean diameter >11 mm. was identified. The LH testing was carried out in the early morning before the TU. When the endometrial thickness reached 7 mm. and the dominant follicle was 16–20 mm. in diameter, patients were considered ready for planning embryo transfer. In patients with no positive urinary LH test despite a follicle of 16–20 mm and endometrial stripe of 7 mm. or more, 5,000 units of urinary hCG (Gonasi HP, Ibsa Italy) were administered. Embryo rewarming and transfer was planned 7 days after the spontaneous LH peak or HCG administration.

Hormonal replacement cycles (AR-FET) consisted of oral estradiol valerate (E2V, 6 mg.) (Progynova, Bayer, Schweiz, AG, 2 mg.) from the second day of the menstrual cycle until the endometrial thickness reached at least 7 mm. The embryo transfer was scheduled after 5 days from the progesterone start, continuing the same estradiol dose. If endometrial thickness was less than 7 mm. after 12 days of E2V, the dose was increased to 8 mg./day. Endometrial preparation for transfer consisted of continued estradiol (6–8 mg. a day E2V) combined with 600 mg of vaginal micronized progesterone tablets (Prometrium, Rottapharm S.p.a., or Progeffik, Effik Italia S.p.a., 200 mg every 8 h) or 180 mg of micronized progesterone vaginal gel (Crinone 8%, Merk, Serono, 90 mg twice a day). Exogenous progesterone supplementation was also started on the day of embryo transfer in the NC-FET group and 2 days after hCG administration in the mNC-FET group using 200 mg. vaginal micronized progesterone tablets (Prometrium, Rottapharm S.p.a., or Progeffik, Effik Italia S.p.a., 200 mg every day) or 90 mg. micronized progesterone vaginal gel (Crinone 8%, Merk, Serono, 90 mg. once a day). Cycles with premature LH surge, poor follicular development, inadequate endometrial thickness or post warming blastocyst degeneration were excluded from the analysis.

Frozen blastocysts were rewarmed on the day of ET and only viable blastocysts were transferred.

ETs were performed using soft catheters under transabdominal ultrasound guidance, according to a pre-determined standardized pre-load technique (27).

Pregnancy tests (serum beta hCG) were obtained 12 after ET and if positive, beta hCG levels were monitored every 48 h until they reached at least 1.000 IU/ml. Transvaginal US was performed 2 weeks later to determine the number of gestational sacs and fetal viability.

Patients continued progesterone supplementation and estradiol in AR-FET until week 12 of gestation.

### Statistical Analysis and Variable Description

Clinical pregnancy was defined as indicated by WHO-ICMART Consensus and the ESHRE register: a pregnancy diagnosed by ultrasonographic visualization of one or more gestational sacs or definite clinical signs of pregnancy. It therefore included ectopic pregnancy. In our center, clinical pregnancy was defined only for bHCG levels of at least 1,000 mIU/ml or clinical and/or ultrasound diagnosis of ectopic implantation and otherwise considered negative (biochemical pregnancies). Live birth was defined as delivery of a living baby after at least 24 weeks of gestation (28, 29). Data were described as number and percentage, or mean and standard deviation, as appropriated. Adherence to Gaussian distribution for continuous variables was verified with Shapiro Wilks test. Association of variable with type of protocol were explored with chi square test, with Fisher correction if necessary, or Kruskal Wallis test, as appropriated. For association with pregnancy and pregnancy outcome we also explore which type of protocol was different, with *post hoc* analyses; all *p*-values were submitted to Bonferroni correction. All possible risk factors for LBR were submitted to univariable logistic regression analysis. Factors with *p*-value under 0.25 entered a subsequent multivariable logistic regression analysis. These results were corrected also for year in which the ET was performed. In the multivariable analysis, only ages of women at the time of embryo freezing and not ages at the time of transfer were included, because not considered relevant (30, 31). Also, interactions between variables used in the multiple regression were considered, but none of them were statistically significant. A *p*-value under 0.05 was considered as significant. All analyses were conducted using Stata Statistical Software: Release15 (College Station, TX: StataCorp LLC).

## Results

The analysis included 2,895 cycles. Patients were divided into three groups: group I (NC-FET, *n* = 561), group II (mNC-FET, *n* = 1,749) and group III (AR-FET, *n* = 585). Patients demographic and clinical characteristics among the three groups are reported in Table 1.

**Table 1:** Patients' demographic and clinical characteristics (all *p*-values comparisons Bonferroni corrected).

	**Natural cycles- frozen–thawed embryo transfer (NC-FET)**	**Modified natural cycles- frozen–thawed embryo transfer (mNC-FET)**	**Artificial replacement cycles- frozen–thawed embryo transfer (AR-FET)**	***p***	***p*** **NC-FET vs. mNC-FET**	***p*** **NC-FET vs. AR-FET**	***p*** **mNC-FET vs. AR-FET**
Number of cycles	561	1,749	585				
Women's age at blastocyst vitrification.	35.4 ± 4.3	35.3 ± 4.0	34.4 ± 4.2	0.0001	1.0000	0.0003	<0.0001
Body Mass Index (kg/m^2^)	21.8 ± 3.0	21.8 ± 3.0	22.5 ± 3.3	0.0001	1.0000	0.0001	<0.0001
Duration of infertility (months)	55.7 ± 30.9	54.5 ± 43.6	58.2 ± 33.5	0.0034	0.6080	0.3584	0.0025
Follicle Stimulating Hormone (FSH)(mIU/mL)	7.09 ± 2.50	6.85 ± 2.44	6.26 ± 2.47	0.0001	0.1528	<0.0001	<0.0001
Anti-Mullerian Hormone (AMH) (ng/mL)	2.87 ± 2.30	3.31 ± 2.58	5.12 ± 4.36	0.0001	0.0005	<0.0001	<0.0001
PCOS	28 (4.99%)	118 (6.75%)	140 (23.93%)	<0.001	0.411	<0.001	<0.001
**Infertility causes**							
Male	207 (36.90%)	709 (40.54%)	213 (36.41%)	0.109			
Idiopathic	73 (13.01%)	249 (14.24%)	48 (8.21%)	0.001	1.000	0.024	<0.001
Disovulatory	7 (1.25%)	31 (1.77%)	63 (10.77%)	<0.0001	1.000	<0.001	<0.001
Endometriosis	27 (4.81%)	63 (3.60%)	24 (4.10%)	0.428			
Mixed: male and female	84 (14.97%)	268 (15.32%)	97 (16.58%)	0.711			
Multiple	27 (4.81%)	65 (3.72%)	22 (3.76%)	0.494			
Tubal	74 (13.19%)	226 (12.92%)	88 (15.04%)	0.422			
Reduced ovarian reserve	61 (10.87%)	130 (7.43%)	26 (4.44%)	<0.0001	0.030	<0.001	0.037
Other (genetic or multiple miscarriages)	1 (0.18%)	8 (0.46%)	4 (0.68%)	0.440			
Freeze all	127 (22.64%)	472 (26.99%)	256 (43.76%)	<0.001	0.123	<0.001	<0.001
Number of previous pregnancies obtained from fresh embryo transfer	89/417 (21.34%)	260/1191 (21.91%)	67/294 (22.79%)	0.898			

Women average age at embryo freezing was 35.4 ± 4.3 years for NC-FET, 35.3 ± 4.0 years for mNC-FET and 34.4 ± 4.2 years for AR-FET (*P* = 0.0001). Average basal Follicular Stimulating Hormone (FSH) values were 7.09 ± 2.50 mIU/mL in NC-FET, 6.85±2.44 mIU/mL in mNC-FET) and 6.26 ± 2.47 mIU/mL in AR-FET (*P* = 0.0001). The average value of Anti-Mullerian Hormone (AMH) was 2.87 ± 2.30 ng/mL in NC-FET, 3.31±2.58 ng/mL in mNC-FET and 5.12 ± 4.36 ng/mL in AR-FET (*p* = 0.0001). The mean value of Body Mass Index (BMI) was 21.8 ± 3.0, 21.8 ± 3.0, and 22.5 ± 3.3 kg/m^2^ (*p* = 0.0001) in the three groups, respectively. A significant difference was found among the 3 groups in the percentage of anovulatory disorders, idiopathic and reduced ovarian reserve as main indication to treatment (Table 1). The number of polycystic ovary patients was significantly higher (<0.001) in the AR group (23.93%) than in NC (4.99%) and mNC (6.75%) as well as the number of freeze all cycles was significantly higher (43.76%) in the AR group than in NC (22.64%) and mNC (26.99%).

The CPR in NC-FET was 32.62% (183/561), in mNC-FET was 43.05% (753/1,749) and in AR-FET was 37.26% (218/585); the LBR in NC-FET was 24.06% (135/561), in mNC-FET was 33.56% (587/1,749) and in AR-FET was 25.81% (151/585). Both CPR and LBR were significantly higher in the mNC- FET group (Table 2). A statistically significant (*p* < 0.001) higher LBR for mNC-FET vs. NC-FET (OR 0.49–0.78) and AR-FET (OR 0.47–0.74) was observed.

**Table 2:** Pregnancy outcomes (all *p*-values comparisons Bonferroni corrected).

	**Natural cycles- frozen–thawed embryo transfer (NC-FET)**	**Modified natural cycles- frozen–thawed embryo transfer (mNC-FET)**	**Artificial replacement cycles- frozen–thawed embryo transfer (AR-FET)**	***p***	***p*** **NC-FET vs. mNC-FET**	***p*** **NC-FET vs. AR-FET**	***p*** **mNC-FET vs. AR-FET**
Number of cycles	561	1,749	585				
Clinical Pregnancy Rate	183 (32.62%)	753 (43.05%)	218 (37.26%)	<0.001	<0.001	0.298	0.042
Biochemical Pregnancies	21 (3.74%)	61 (3.49%)	26 (4.44%)	0.572			
Live Birth Rate	135 (24.06%)	587 (33.56%)	151 (25.81%)	<0.001	<0.001	1.000	0.001
Stillbirth (%)	1 (0.55%)	2 (0.27%)	0	0.481			
Ectopic pregnancies (%)	6 (3.28%)	3 (0.40%)	4 (1.83%)	0.002	0.009	1.000	0.147
**Miscarriages (%)**							
Before 12 weeks	36 (19.67%)	146 (19.39%)	60 (27.52%)	0.031	1.000	0.198	0.030
After 12 weeks	3 (1.64%)	9 (1.20%)	2 (0.92%)	0.850			
Therapeutic abortion (%)	2 (1.09%)	4 (0.53%)	1 (0.46%)	0.640			
Lost to follow up	0	2 (0.27%)	0	1.000			
Twins (%)	2/135 (1.48%)	5/753 (0.66%)	0	0.217			
Number of babies born	137	592	151				
Baby's weight at birth (kg)	3.13 ± 0.53	3.28 ± 0.55	3.33 ± 0.57	0.001	0.002	0.002	0.785
Weigth over 90th percentile (3.9 Kg)	21 (13.91%)	60 (10.14%)	8 (5.84%)	0.070			
Baby's sex (Male %)	73 (53.28%)	298 (50.34%)	91 (60.26%)	0.091			
Gestational age at delivery (weeks)	38.5 ± 2.1	38.9 ± 2.1	38.7 ± 3.0	0.018	0.015	0.117	1.000

Ectopic pregnancies were 6 (3.28%) in NC-FET, 3 (0.40%) in mNC-FET, 4 (1.83%) in AR-FET (*p* = 0.002) and miscarriages <12 weeks were 36 (19.67%) in NC-FET, 146 (19.39%) in mNC-FET, 60 (27.52%) in AR-FET (0.031).

The number of biochemical pregnancies, miscarriages >12 weeks, therapeutic abortion, monozygotic twins, weight over 90th percentile (3.9 Kg), baby's sex ratio were not significantly different among the 3 groups. Baby's birthweights were 3.13 kg ± 0.53, 3.28 kg ± 0.55, 3.33 kg ± 0.57 (0.001), and the gestational age at delivery were 38.5 ± 2.1, 38.9 ± 2.1, 38.7 ± 3.0 weeks (0.018) respectively in NC-FET, mNC-FET and in AR-FET (Table 2).

Both univariable and multivariable analyses and results corrected for the main confounding factors (women age at embryo freezing, BMI, previous seeking pregnancy time, FSH and AMH values) are presented in Table 3.

**Table 3:** Prognostic factors for Live Birth Rate and Odds Ratio (OR) analysis (Data in multiple logistic regression analysis are corrected by years of embryo transfer.

**Live birth**	**Univariable**		**Multivariable**	
	**OR (95% CI)**	***p***	**OR (95% CI)**	***p***
Women's age at embryo freezing	0.93 (0.91–0.95)	<0.001	0.93 (0.91−0.95)	<0.001
Women's age at embryo transfer	0.93 (0.91–0.95)	<0.001		
Body Mass Index	0.98 (0.96–1.01)	0.213	1.00 (0.97–1.03)	0.961
Duration of infertility, months	0.999 (0.997–1.001)	0.391		
Follicle Stimulating Hormone (FSH) (mIU/mL)	0.99 (0.96–1.02)	0.472		
Anti-Mullerian Hormone (AMH) (ng/mL)	1.05 (1.03–1.08)	<0.001	1.04 (1.01 - 1.07)	0.013
PCOS	0.98 (0.75–1.27)	0.864		
Freeze all	1.30 (1.10–1.54)	0.002	0.99 (0.81–1.21)	0.932
Previous pregnancies obtained from fresh embryo transfer	0.92 (0.74–1.15)	0.477		
**Group**				
Natural cycles frozen–thawed embryo transfer (NC-FET)	1		1	
Modified natural cycles frozen–thawed embryo transfer (mNC-FET)	1.59 (1.28−1.98)	<0.001	1.66 (1.39–1.98)	<0.001
Artificial replacement cycles frozen–thawed embryo transfer (AR-FET)	1.10 (0.84−1.44)	0.494	0.95 (0.71–1.27)	0.724

*Year 1.08 (1.01−1.17) p = 0.036)*.

Data in multiple logistic regression analysis were corrected by the year in which the ET was performed to avoid further potential bias. Both the Odds Ratios (OR) of the univariable and multivariable analyses were similar, confirming better pregnancy outcomes for mNC-FET vs. NC-FET (1.59 and 1.66, respectively) and equal probability of AR-FET respect NC-FET (1.10 and 0.95, respectively). Only female age, AMH and mNC-FET vs. NC-FET were statistically significant favorable prognostic factors for LBR (Table 3).

## Discussion

Cryopreservation of exceeding embryos has become a common procedure in Assisted Reproductive Techniques (ART) and is an important tool to assess cumulative delivery rate (32). Improvements in laboratory technologies and techniques have contributed to an increased availability of good quality surplus embryos for vitrification (33, 34) and at the same time have also contributed to the establishment of more rigid guidelines regulating the number of fresh embryos being transferred (35) to limit multiple pregnancies and their well-known associated obstetric complications (36). However, what it is still a matter of debate is the choice of the most optimal preparation protocol for FET. The data until recently don't provide strong evidence in support to the use of mNC-FET in alternative to NC-FET or AR-FET (37).

To our knowledge, this is the first single center European study including a so large case sample (2,895 FET cycles) that directly evaluated the outcome of three preparation protocols: (a) the natural cycle (NC-FET); (b) the modified natural cycle (mNC-FET) using ovulation triggering by hCG; and (c) the artificial replacement (AR-FET) with hormonal preparation of the endometrium. In evaluating single blastocyst transfer, encompassing most of the cycles performed with mNC, our data are in agreement with a recent study of Liu et al. comparing cleavage and blastocyst stage FET in 1,846 patients, with 308 were natural (combining NC and mMC), with a higher LBR and lower abortion than in AR cycles in a young women age population (38).

Many patients and clinics prefer the use of NC protocols (39) since they are relatively simple to implement, are associated with good patient's compliance and avoiding the use of prolonged hormonal supplementation. However, the timing of transfers in NC requires an accurate determination of ovulation. Indeed, natural cycle transfers have high cancellation rates (40) because of the inability to determine the exact time of ovulation. In the present study, the practice of using a single, daily urinary test before US to detect the LH surge, was considered favorable because it is cheaper and less stressful for patients compared to repeating the LH test multiple times during the day or to multiple blood samplings. However, it is less accurate and false positive test results occur in approximately 7% of cases (41). In addition, unlike hormone replacement cycles, the date for embryo rewarming and transfer cannot be programmed, a particularly crucial problem for centers that do not operate 7 days a week. In addition to the above drawbacks, only patients with regular ovulatory cycles can be included in NC–FET programs.

In literature, there are various methods to establish the time of ovulation, considering serum LH elevation (<17 mIU/ml, or 2.5 times higher than previous determinations) considering estradiol drop, and increase in progesterone levels >1 ng /ml. or lower (42). A very recent publication (38) comparing natural and HR cycles when a follicle was > than 17 mm. and serum LH < to 20 mIU / ml followed patients until ultrasound signs of ovulation and scheduling blastocyst transfer 5 days after. The same paper describes a very different luteal phase support. In AR cycles progesterone was started for an endometrial thickness of at least 7 mm, progesterone <1.5 ng. /ml scheduled after 6 days after and in NC progesterone started when the follicle collapsed, and the transfer scheduled 5 days after.

Our study suggests there is a higher miscarriage rate with HT cycles than NC and m-NC confirming a very recent report in a younger population (38). Perhaps the absence of the corpus luteum as in AR-FET can play an important role not only as e a factor predisposing women to develop preeclampsia (43), but also in predisposing first trimester losses.

The present study, however, provides evidence that triggering ovulation with hCG (mNC–FET) is an efficient protocol in terms of CPR and LBR than either serial monitoring for ovulation detection in patients undergoing NC-FET or in patients using hormonal support for ET (AR–FET). Moreover, since ovulation triggering by hCG significantly reduces the number of monitoring visits that are necessary to schedule the day for frozen embryo transfer, this approach may also be more favorable in terms of patient convenience and cost–effectiveness of the entire cycle.

At present however, it is not possible to determine whether these results are due to the direct effect of hCG on luteal phase or to the fact that mNC-FET provides a more correct timing from the urinary LH peak to the embryo transfer as compared to a single urinary LH kit whose reliability is still questionable (41).

Proper timing of embryo transfer in frozen/rewarmed cycles is one of the most crucial steps for the successful outcome. Among the three preparation protocols considered, our data showed that the use of mNC is associated with the highest CPR and LBR reflecting an optimal synchronization between endometrial growth and embryonic development.

Some of the limits of this study are related to the recruitment period spanning over 7 years (from 2011 to 2017) during which small variations in laboratory techniques might have impacted on the final reproductive outcome.

Therefore, a *post hoc* study was implemented to compare different reproductive outcomes over the period 2011–2017. Indeed, in the latest years of this retrospective analysis a larger use of mNC-FET vs. pure NC-FET or AR-FET was chosen in daily clinical practice at our Center due to the more favorable outcomes observed in a preliminary analysis carried out on a partial dataset.

*Post hoc* univariate and multivariate analysis for prognostic factors for LBR showed a statistical significantly relation only for female age, AMH and mNC-FET in comparison with NC-FET, but not with AR-FET.

A further *post hoc* data analysis may be useful to verify if the three different population groups differed for other prognostic factors not considered in the study. However, the use of multivariate *post hoc* analyses could be a more confounding factor reducing the evidence even of large real-world data (44).

To conclude, a prospective randomized study should be carried out to corroborate the data presented here and propose mNC as the best replacement protocol during FET cycles.

## Data Availability Statement

The datasets for this study can be found in the Humanitas repository. Due to our internal policy, no raw data are available for external use. The datasets generated for this study are available on request to the corresponding author.

## Ethics Statement

The studies involving human participants were reviewed and approved by Humanitas Ethic Board. The patients/participants provided their written informed consent to participate in this study.

## Author Contributions

PL designed the project, collected data, and drafted the manuscript. FC, RD, VC, CR, AB, AS, and EA collected clinical data. EM analyzed clinical data and performed the statistical analysis. PP revised the manuscript. All authors participated to final manuscript.

## Conflict of Interest

The authors declare that the research was conducted in the absence of any commercial or financial relationships that could be construed as a potential conflict of interest.
